# Study of Electroless-Deposited Zn on the Surface of Mg-Li Alloy

**DOI:** 10.3390/ma16165511

**Published:** 2023-08-08

**Authors:** Anyu Yue, Yong Cao, Yi Zhang, Shenggang Zhou

**Affiliations:** Faculty of Materials Science and Engineering, Kunming University of Science and Technology, Kunming 650093, China; yaykmust@126.com (A.Y.); caoyongkmust@126.com (Y.C.); zyykmust@126.com (Y.Z.)

**Keywords:** Mg-Li alloy, electroless-deposited, zinc coating, corrosion resistance

## Abstract

The Mg-Li alloy stands as the lightest metallic structural material known to date, finding a wide range of applications. However, its development has been hindered by its susceptibility to oxidation and corrosion. In this study, we aimed to address this issue by employing electroless deposition to form a protective zinc layer on the surface of a magnesium–lithium alloy. The optimization of the zinc layer was achieved through varying parameters such as the zinc dipping time (1~10 min), temperature (20~70 °C), and zinc content (20~200 g/L). Surface characterization was performed using scanning electron microscopy (SEM) and X-ray diffraction, while electrochemical tests and scratch tests were conducted to evaluate corrosion resistance and coating adhesion. The results demonstrated the successful formation of a uniform and dense pure zinc layer on the surface of the Mg-Li alloy when the zinc-dipping time was set at 5 min, the temperature was at 30 °C, and the zinc content was at 50 g/L. Under these conditions, the corrosion potential of the Mg-Li alloy experienced the greatest positive shift, reaching as high as −1.38 V. Additionally, the corrosion current was minimized, measuring at 2.78 × 10^−6^ A/cm^2^. Furthermore, the maximum arc tolerance radius was observed. Consequently, the electroless deposition of zinc onto Mg-Li alloys significantly improves their corrosion resistance and bonding, opening up new prospects for the application of zinc-plated Mg-Li alloys.

## 1. Introduction

The Mg-Li alloy currently holds the distinction of being the lightest metallic structural material known. With a density of less than 1.5 g/cm^3^, it is 10~30% lighter than standard magnesium alloy, making it a crucial component in the realm of ultralight materials [1]. The Mg-Li alloy offers numerous advantages, including high specific strength and stiffness, a small elastic modulus, good machining performance, resistance to electromagnetic interference, exceptional shock absorption, effective electromagnetic shielding, and minimal environmental impact [2,3,4]. As a result, it holds significant potential for a wide range of applications, particularly in the aerospace and defense industries [5,6,7].

However, the corrosion resistance of Mg-Li alloys is notably poor due to the negative standard potential of magnesium and the presence of reactive lithium metal [8,9,10]. To fully exploit its exceptional properties, effective surface protection measures must be employed to improve the corrosion resistance of Mg-Li alloys [10,11,12,13]. Currently, various surface treatments, including anodization, ion implantation, nitridation, electroplating, and electroless deposition are used to address weaknesses such as limited room temperature plasticity, low strength, reactivity, and susceptibility to reactions [14,15]. However, some of these methods suffer from drawbacks such as complex processes, high costs, and environmental pollution [16,17,18]. Organic coatings can improve corrosion resistance, frictional wear properties, and aesthetics [19]. In addition, powder coating is a viable approach for forming organic coatings on magnesium alloys. Electrochemical polymerization enables the formation of organic polymer coatings. Nevertheless, organic coatings may present challenges such as non-conductive film layers and environmental concerns [20]. Consequently, research efforts have been focused on developing surface treatment technologies for magnesium and its alloys that meet environmental requirements while offering commercial value. Electroless deposition stands out as a method with low cost, high efficiency, and simplicity. While initially used primarily for mechanical improvements, further research has shown that electroless deposition not only enhances mechanical properties but also corrosion resistance [21]. In Eman’s study [22], the material exhibited superior morphology, structure, roughness, wettability, hardness, and corrosion resistance after heat treatment compared to conventional NiP-plated antibacterial and corrosion-resistant coatings. Moreover, electroless deposition is considered a relatively environmentally friendly method [23,24].

The traditional zinc leaching process typically necessitates high-temperature conditions, leading to limited usability and an overall requirement for elevated temperatures. Moreover, this process results in long zinc leaching times and unevenly obtained zinc coatings. Therefore, there is a significant need to explore an effective method for preparing a zinc-dipped layer at room temperature within a shorter timeframe. In a study by Luo [17] et al., Mg-Li alloy sheets were pretreated and subjected to a process involving a NiCO_3_-2Ni(OH)_2_-4H_2_O solution to create a thin Ni-P alloy film, followed by plating in a NiSO_4_-6H_2_O solution to generate a protective layer. The research demonstrated that the coating exhibited favorable corrosion resistance. Wu [11] et al. employed the electroplating method on the surface of a copper-coated Mg-Li alloy, resulting in improving corrosion and wear resistance properties. Thus, the electroless deposition of Mg-Li alloys significantly impacts both the material protection and subsequent processing of Mg-Li alloys through zinc dipping.

This article focuses on the development of a zinc dipping solution incorporating organic acid complexing agents to form a zinc dip layer on the surface of Mg-Li alloys, thereby enhancing their corrosion resistance. Unlike conventional zinc dipping methods, this process requires less time, lower temperatures, and produces a denser zinc dip layer, thereby improving the performance of subsequent applications. The effect of zinc leaching on the surface of Mg-Li alloys was investigated by employing an electrochemical analyzer, scanning electron microscopy (SEM), X-ray diffraction (XRD), and coating adhesion tests. Different leaching times, zinc contents, and temperatures were examined to assess their impact on Mg-Li alloy surfaces.

## 2. Materials and Methods

### 2.1. Electroless Plating of Zn

**Figure 1:**
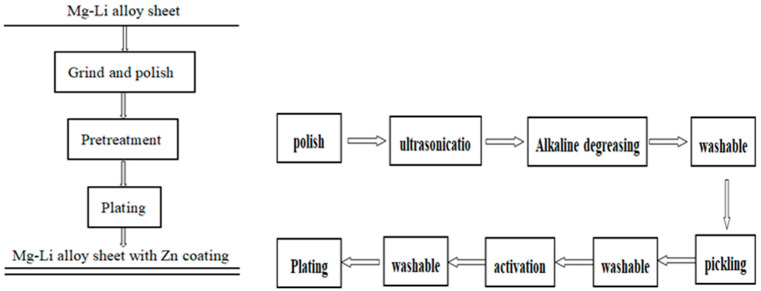
Process diagram for electroless Zn plating on a Mg-Li alloy sheet.

The specific process, illustrated in Figure 1, involved the following steps: the Mg-Li alloy (Li_3_Mg_7_) was initially cut into sheets measuring 10 mm × 10 mm × 5 mm, followed by grinding and polishing. After the polishing stage, the samples underwent a series of pretreatments, including ultrasonication, alkaline degreasing, pickling, activation, and chemical zinc dipping. The grease removal process employed saponification of saponified grease by caustic solution and emulsification of unsaponified grease through surface activators. An acid solution with a specific composition and process conditions was utilized to induce a chemical reaction, wherein the mucus film thickness on microscopic bulges was minimized or the passivation film was activated to preferentially dissolve the microscopic bulges while minimizing the dissolution of microscopic depressions. This resulted in the elimination of surface protrusions, rendering the surface flat and smooth, thereby revealing the crystalline structure of the matrix texture. The activation step aimed to further remove oxides and hydroxides, exposing the matrix weave fully and facilitating the formation of the zinc layer during the zinc dipping process. Distilled water was used to clean the intervals between each process. Table 1 presents the chemical composition of each treatment. All chemicals utilized in the study were of chemical purity and were sourced from reputable suppliers such as Macklin and Aladdin.

The specific operations are as follows:

- Alkaline degreasing: The alkaline wash solution was heated to 70 °C and the sonicated samples were immersed in this solution for 6 min. Afterward, the samples were immediately rinsed with hot distilled water to prevent the generation of insoluble silicic acid during the subsequent steps. Finally, the samples were rinsed with cold distilled water.
- Acid pickling: The acid wash solution was heated to 30 °C, and the alkaline washed samples previously subjected to alkaline degreasing were immersed in the solution for 45 s. Throughout the cleaning process, the sample was continuously shaken to prevent over-etching of the surface. Subsequently, the samples were thoroughly rinsed with an abundant amount of distilled water.
- Activation: Acid activation was used in this experiment at room temperature. The samples were immersed in the activation solution for 45 s, followed by a thorough rinse with distilled water.
- Immersion in the zinc bath: The samples were immersed in the zinc bath for 1–10 min at a temperature ranging from 20–70 °C. After immersion, the samples were extensively rinsed with distilled water and dried using cold air.

### 2.2. Coating Characterization

The microstructure of the zinc dip layer and the cross-section of the layer were examined using SEM (TESCAN VEGA COMPACT). The composition of the coating was determined through X-ray energy spectrometry (EDS) combined with SEM. The accelerating voltage ranged from 10 to 25 kV, and the beam spot diameter was 2 to 3 mm. The phase identification of the electrodeposit was carried out by an X-ray diffractometer (XRD-7000). Cu-Kα and 2θ/θ scanning modes were employed to characterize the deposition patterns within the range of 20~80°. The scanning speed was set at 5°/θ, and the scanning angle was set at 0.01°.

### 2.3. Electrochemical Testing

The electrochemical properties of the zinc-dipped Mg-Li alloy were evaluated using the Corrtest electrochemical test system. To ensure 1 cm^2^ exposure to the electrolyte, the samples were cut into dimensions of 10 mm × 10 mm × 5 mm. The working electrode was connected to a copper wire by the indium press method, which minimized connection resistance and avoided any interference with the electrochemical properties. The joint between the copper wire and the magnesium alloy plate was then isolated from the sample using an epoxy working surface to prevent electrolyte penetration and its impact on the test results. The electrochemical properties of zinc layers were analyzed using a three-electrode system in the CORTEST electrochemical test system. The working electrode consisted of the specimen, the auxiliary electrode was a platinum sheet, and the reference electrode was a saturated calomel electrode. The electrolyte used was a 3.5 wt.% NaCl solution (as the corrosion rate of magnesium alloy in 3.5 wt.% to 5 wt.% NaCl solution remains relatively stable). The working electrode area was set at 1 cm^2^, and the three-electrode system was wrapped in a copper mesh to act as a shield. In the kinetic potential scanning experiments, the samples were initially immersed in the electrolyte for 10 min to stabilize the open circuit potential (OCP), *E*_0_. The scan rate for all measurements was 10 mV/s.

## 3. Results and Discussion

### 3.1. Zinc Deposition Process

**Figure 2:**
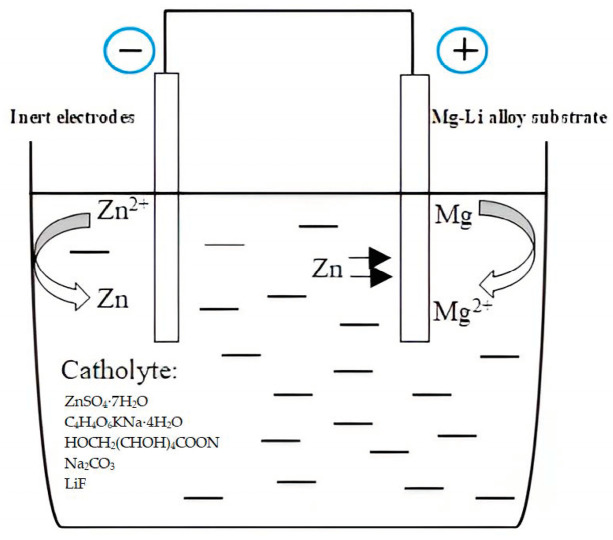
Schematic diagram of the galvanic cell.

The majority of zinc present in the zinc-deposited solution exists in the form of complexes [25]. The main process involved in the zinc deposition reaction is the replacement reaction between the active magnesium atoms and the Zn^2+^ on the surface of the Mg-Li alloy substrate. As shown in Figure 2, the magnesium atoms lose electrons and form Mg^2+^, while Zn^2+^ gains electrons and deposits as grains on the surface of the magnesium–lithium alloy. The consumption of Zn^2+^ facilitates the dissociation of zinc complexes, ensuring continuous generation of new Zn^2+^, which is essential for the substitution reaction to take place. This reaction leads to the formation of a uniform and dense layer of zinc dip over a certain period of time. The zinc electroless reaction proceeds as follows:

Anode (matrix phase):

(1) $${\mathrm{Mg}}^{0}\to {\mathrm{Mg}}^{2+}+2\;e$$

Cathode (second phase):

(2) $${\left[\mathrm{ZnLx}\right]}^{n}\to {\mathrm{Zn}}^{2+}{+\mathrm{xL}}^{(n-2)/2}$$

(3) $${\mathrm{Zn}}^{2+}+2e\to {\mathrm{Zn}}^{0}$$

Overall reaction:

(4) $${\mathrm{Mg}+\mathrm{Zn}}^{2+}\to {\mathrm{Mg}}^{2+}+\mathrm{Zn}$$

### 3.2. Microstructural Characteristics

**Figure 3:**
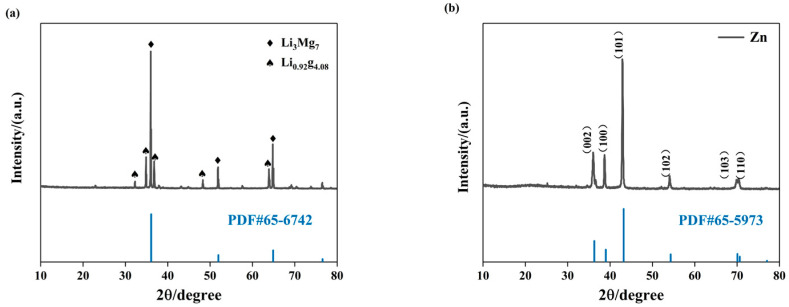
The XRD patterns of the electroless Zn deposition on the Mg-Li alloy: (**a**) LA91 Mg–Li alloy substrate and (**b**) electroless deposition on the layer.

The composition of the dipping zinc layer was analyzed using X-ray diffraction. Figure 3a shows the X-ray diffractogram of the original specimen, whereas Figure 3b depicts the X-ray diffractogram of the specimen after zinc dipping. A comparison between Figure 3a, and Figure 3b reveals the disappearance of certain peaks in the diffractogram after the electroless galvanization of the original sample, indicating the formation of a coating on the surface of the Mg-Li alloy. As illustrated in Figure 3b, the surface composition of the magnesium alloy predominantly consists of zinc. The presence of zinc, characterized by its dense hexagonal structure, is evident, whereas no signals corresponding to the Mg-Li alloy matrix are observed in the pattern. This suggests that the zinc coating is sufficiently dense to provide almost complete coverage of the sample.

**Figure 4:**
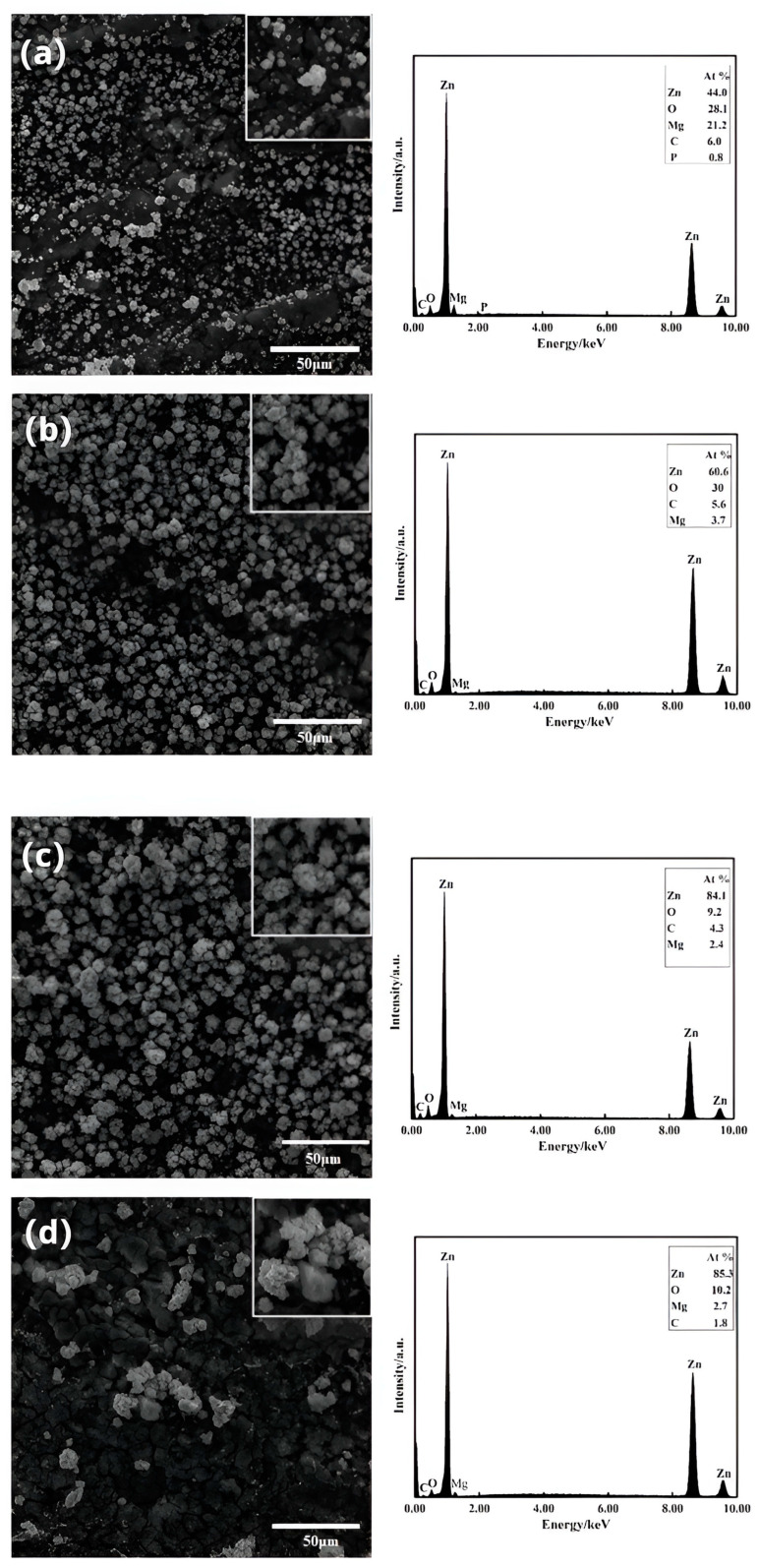
SEM images of dipping layers at different Zn dipping times and their EDS spectra: (**a**) 1 min; (**b**) 3 min; (**c**) 5 min; (**d**) 10 min.

Figure 4 demonstrates the surface morphology of the zinc-dipped layer on the Mg-Li alloy at different zinc dipping times: 1 min, 3 min, 5 min, and 10 min, as shown in Figure 4a–d, respectively. The observations revealed that the zinc dipping process involves the continuous nucleation and growth of zinc grains on the surface. In Figure 4a, zinc grains are observed to nucleate on the surface of the Mg-Li alloy, forming spherical shapes. However, the distribution appears relatively sparse and does not provide complete coverage of the matrix. As the dipping time increases to 1~3 min, the grains grow in size, change shape, and gradually cover the matrix. At this stage, the zinc grains primarily experience numerical growth. When the dipping time reaches 5 min, the substrate is mostly covered. However, extending the dipping time to 10 min results in the uneven growth of zinc grains, with some becoming excessively large. Thus, the surface uniformity of the zinc-dipped layer decreases, along with a decrease in coverage over the Mg-Li alloy. Therefore, the most homogeneous and dense zinc-dipped layer is achieved with a dipping time of 5 min.

Additionally, qualitative analysis of the Mg-Li alloy zinc-dipped layer was conducted using an electron energy spectrometer (EDS) at different zinc dipping times. The results indicate that zinc is the primary component of the coating layer, while small amounts of carbon and oxygen are also present, with magnesium being the base component. The presence of carbon on the surface is associated with the formation process of the zinc layer. In summary, zinc constitutes the main component of the Mg-Li alloy surface, and its percentage increases with time.

**Figure 5:**
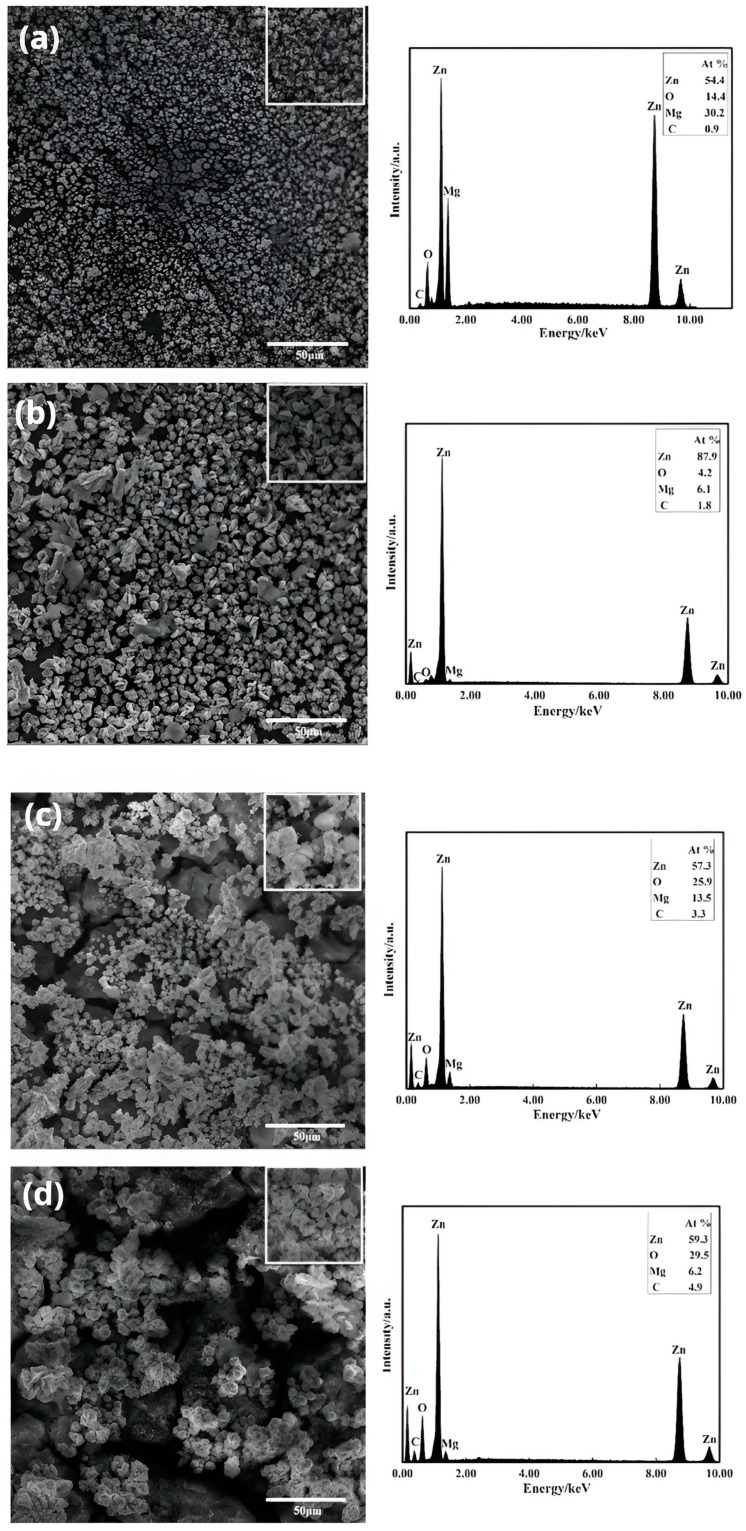
SEM images of dipping layers and their EDS spectra at different zinc contents: (**a**) 20 g/L; (**b**) 50 g/L; (**c**) 100 g/L; (**d**) 200 g/L.

Figure 5 shows the surface morphology of the Mg-Li alloy dipping layer after zinc dipping with varying zinc contents of 20 g/L, 50 g/L, 100 g/L, and 200 g/L, as depicted in Figure 5a–d, respectively. From the graphs, it can be observed that the coating coverage improves as the zinc content increases from 20 to 200 g/L. However, analysis of the EDS plots reveals that at a zinc content of only 20 g/L, the zinc element constitutes only 54.4%, while the magnesium element constitutes 30.2%. This indicates that the zinc coating thickness is thin and that the growth of zinc is incomplete. As the zinc content rises to 50 g/L, grain growth becomes more uniform, the matrix is essentially covered. Furthermore, the zinc element content significantly increases compared to 20 g/L. However, when the content reaches 100~200 g/L, although the zinc content reaches its highest point, the zinc grains pile up to form larger zinc grains with uneven growth. As a result, the coverage of the zinc-dipped layer becomes less dense, and the coverage rate decreases. Therefore, the optimal zinc dipping effect is achieved with a zinc content of 50 g/L.

**Figure 6:**
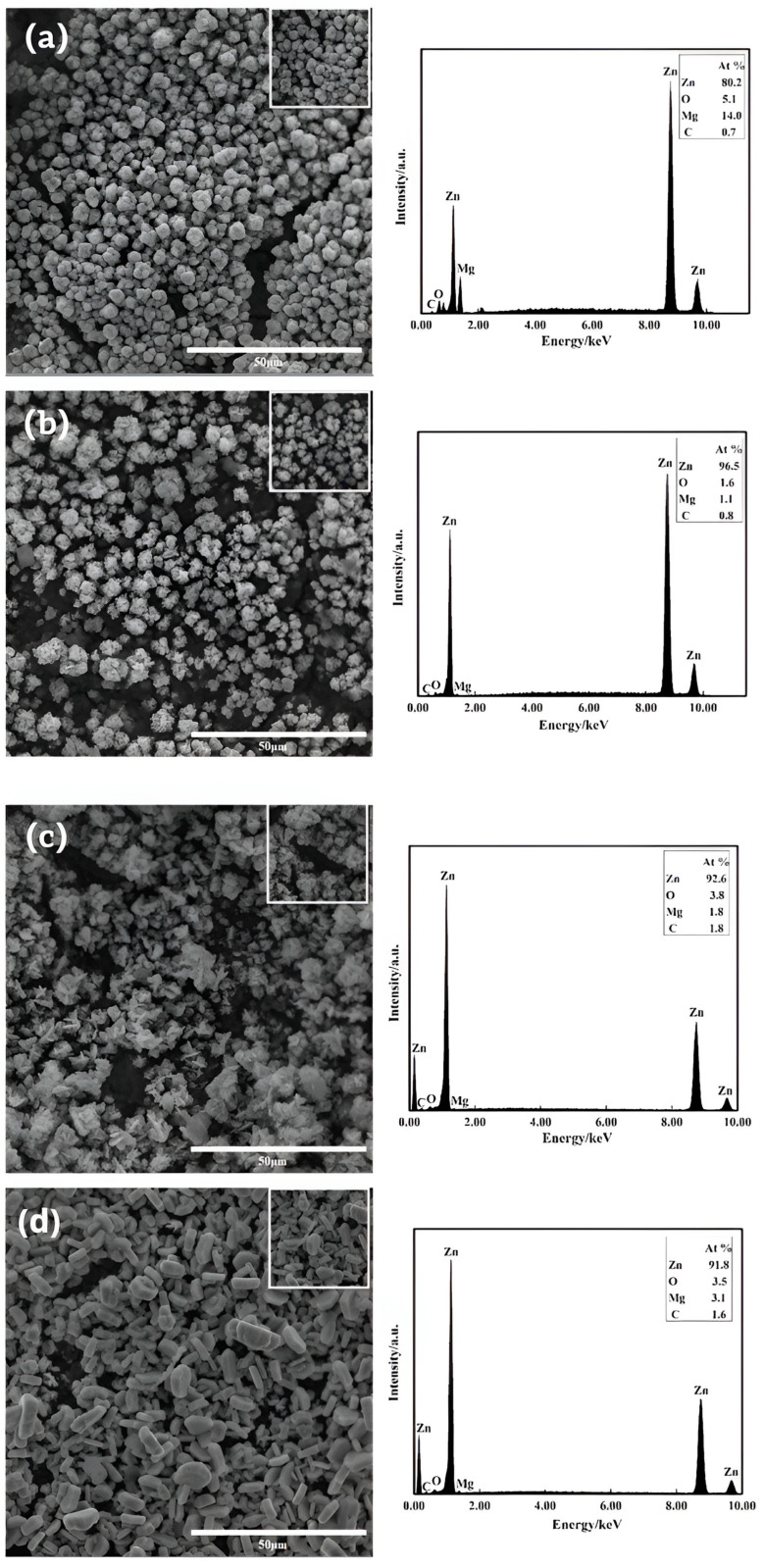
SEM images of the dipped zinc layers at different dipping temperatures and their EDS spectra: (**a**) 20 °C; (**b**) 30 °C; (**c**) 50 °C; (**d**) 70 °C.

Figure 6 presents the surface morphology of the zinc-dipped layer on the Mg-Li alloy at different zinc dipping temperatures: 20 °C, 30 °C, 50 °C, and 70 °C, as shown in Figure 6a–d. It can be seen that at 20 °C, zinc exists as the formation of spherical zinc grains. However, upon comparing the EDS images, it is evident that the coverage of the alloy is incomplete compared to the other temperatures. When the zinc dipping temperature reaches 30 °C, small and flaky zinc grains are uniformly generated over a large area, effectively covering the substrate. Beyond 50 °C, larger and flakier zinc grains are observed, and the gap between the grains increases. Upon reaching 70 °C, the grains become coarser with increased depth. The EDS analysis demonstrates relatively consistent coverage of the Mg-Li alloy matrix; however, the shape of the grains undergoes significant changes, which can affect the properties of the Mg-Li alloy. 

**Figure 7:**
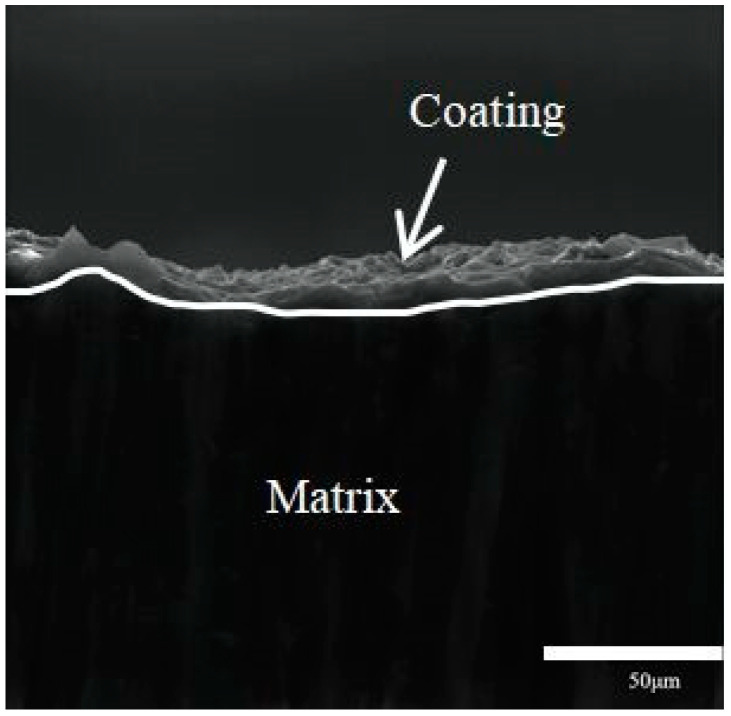
SEM patterns of the Zn deposition cross-section.

Considering the three influencing factors, the optimal conditions for zinc dipping were determined as follows: a dipping time of 5 min, a temperature of 30 °C, and a zinc ion content of 50 g/L. The cross-sections of the material were examined under these conditions. Figure 7 illustrates the cross-section morphology of the Mg-Li alloy galvanized layer observed by a scanning electron microscope. The coating and substrate exhibit no apparent boundary, and subsequent tests of binding force indicate strong adhesion of the coating. Consequently, the coating provides a certain level of protection for the Mg-Li alloy substrate.

### 3.3. Electrochemical Characterization of Electroplating

**Figure 8:**
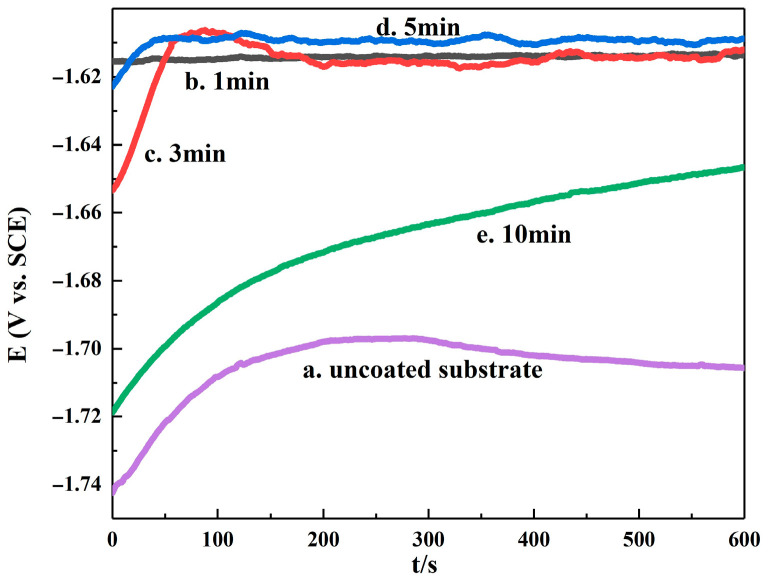
Open circuit potential vs. immersion time curves for the Mg–Li alloy in different Zn dipping times.

The open circuit potential versus time curve for the Mg-Li alloy impregnation process is depicted in Figure 8. A more positive open circuit potential indicates higher material inertia and lower susceptibility to corrosion, while a more negative open circuit potential indicates a tendency to corrode. A smooth open circuit potential curve shows that the corrosion process on the surface of the material and the deposition of corrosion products have reached a stable state. From the diagram, it can be seen that at an impregnation time of 5 min, the potential of the Mg-Li alloy gradually rises and then stabilizes, indicating the formation of a more stable passivation film on the material surface, providing better protection to the substrate. At a dipping time of 3 min, the potential rises rapidly, slightly decreases, and then stabilizes, indicating incomplete corrosion and a relatively active alloy surface. Conversely, at a zinc dipping time of 10 min, the open circuit potential becomes more negative, showing poor corrosion resistance. These findings align with the results from the scan chart. Overall, the open circuit potential graph demonstrates that the corrosion effectiveness, in terms of a stable circuit, follows the order: 5 min > 3 min > 1 min > 10 min > uncoated substrate.

**Figure 9:**
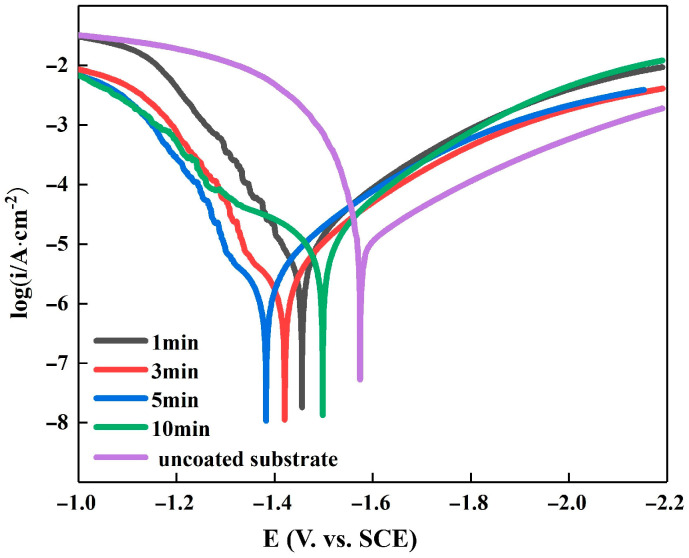
Tafel polarization curves for Zn plating of substrates and samples as affected by the time in solutions containing 3.5% NaCl.

Figure 9 shows the polarization curves of the Mg-Li alloy specimens in a 3.5% NaCl solution at different dipping times. The Tafel fit data obtained from these polarization curves are summarized in Table 2. A more positive self-corrosion potential signifies higher material inertness and better corrosion resistance, while a larger self-corrosion current suggests a faster corrosion rate and poorer corrosion resistance. In a previous study, Sheng et al. [25] reported a self-corrosion voltage and self-corrosion current of −1.428 V and 3.397 × 10^−5^ A/cm^2^, respectively, at a zinc dipping time of 5 min. In this experiment, the polarization curve shifted the most, and the corrosion potential reached −1.386 V at a zinc immersion time of 5 min. After zinc immersion, the corrosion current density was lower than that of the Mg-Li alloy matrix. However, with the increasing zinc immersion time, the current density initially decreased and then increased. The minimum current density of 4.071 × 10^−6^ A/cm^2^ was observed at a zinc dipping time of 5 min, which was an order of magnitude lower than the billet samples. Moreover, the anodic and cathodic slopes were at their maximum, indicating the highest resistance to corrosion on the surface material. Thus, the samples exhibited high inertness and corrosion resistance at 5 min, with potentials elevated and currents decreased by an order of magnitude compared to the previous experiments. The negative shift in corrosion potential and increase in corrosion current at 5 min is due to the reduced surface uniformity of the zinc-dipped layer and the decreased coverage of the Mg-Li alloy, resulting in reduced corrosion resistance. Conversely, the positive shift in corrosion potential and decrease in corrosion current density show the protective effect of the zinc-dipped layer on the Mg-Li alloy [26]. The most positive corrosion potential and the lowest corrosion current density were observed at 5 min, indicating that the zinc-dipped layer was the most uniform and dense at this time.

**Figure 10:**
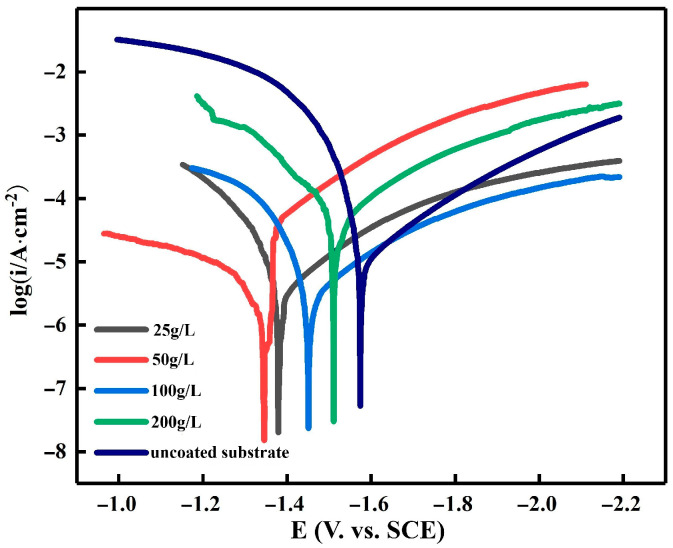
Tafel polarization curves for Zn plating of substrates and samples as affected by Zn content in solutions containing 3.5% NaCl.

**Figure 11:**
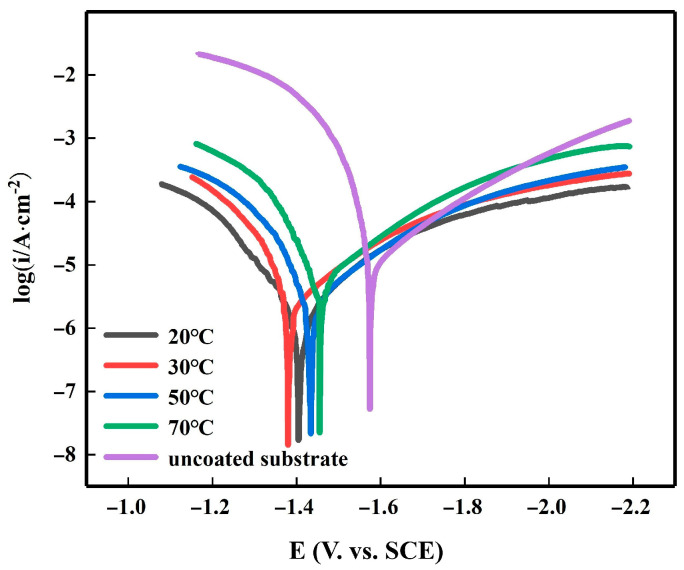
Tafel polarization curves for the Zn plating of substrates and samples as affected by temperature in solutions containing 3.5% NaCl.

Figure 10 shows the polarization curves of the Mg-Li alloy specimens immersed in a 3.5% NaCl solution with varying zinc contents. Table 3 summarizes the Tafel fit data, which reveals the results obtained from analyzing the polarization curves of the five samples. In Figure 10, it is evident that at a zinc content of 50 g/L, the overall alloy exhibits a notable rightward shift, reaching a value of −1.316 V. It is worth noting that the corrosion resistance improves as the corrosion potential becomes more positive. Apart from the matrix sample, the corrosion potential of the alloy curve at a zinc content of 200 g/L is the most negative, indicating a more severe corrosion effect. Combining this information with the data provided in Table 3, we observe that the minimum current density of 2.783 A/cm^2^ is obtained at 50 g/L. Therefore, the most positive self-corrosion potential and the lowest self-corrosion current can be attained at a zinc content of 50 g/L. Furthermore, when considering the SEM plots of the different contents, it becomes evident that the reduction in the surface uniformity of the zinc dip layer and the coverage of the Mg-Li alloy at 50 g/L exhibit exceptional performance compared to the other contents. This indicates a high level of corrosion resistance. Moving on to the impact of temperature on the polarization curves of the Mg-Li alloy dipped in zinc, Figure 11 demonstrates this relationship, while Table 4 provides the Tafel fit data for the polarization curves. The data reveal that the most positive corrosion potential and the smallest corrosion current curves are obtained at a temperature of 30 °C, with values of −1.404 V and 2.818 A/cm^2^ respectively. The cathodic-anodic slopes for this temperature are 360.41 mV and 430.06 mV, respectively. However, upon comparing the individual curves, it can be seen that they do not exhibit significant changes at each temperature relative to the other two variables. This lack of variation can be attributed to the minimal alteration in zinc content observed at each temperature, as indicated by the EDS analysis. Consequently, the influence of temperature on the Mg-Li alloy zinc dip process is considered insignificant.

**Figure 12:**
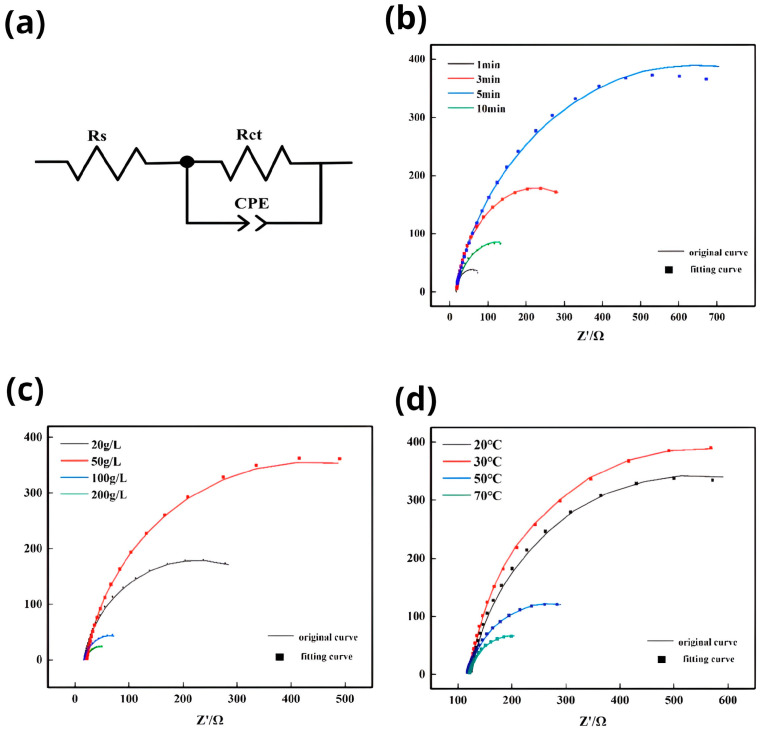
(**a**) equivalent circuit diagram; (**b**) Influence of zinc leaching time on impedance spectrum; (**c**) influence of zinc content on impedance spectra; (**d**) influence of zinc leaching temperature on impedance spectrum.

Electrochemical impedance spectroscopy (EIS) is a rapid, non-destructive, and highly sensitive testing method that effectively characterizes the corrosion behavior and degradation tendencies of materials. Equivalent circuits (ECs) are commonly employed to propose models or mechanisms that elucidate the corrosion process. In Figure 12a, the equivalent circuit for the corrosion process is depicted. It consists of several components: Rs represents the solution resistance, Rct denotes the charge transfer resistance, and CPEc(Q) is a constant phase angle element that approximates the double-layer capacitance at the interface between the study electrode and the solution. Utilizing the formula $Z=R_{s}+\frac{R_{ct}}{1+j\omega R_{ct}}$, the arc of capacitive reactance expands diagonally upward and to the right, with its resistance value increasing. Figure 12b shows the electrochemical impedance spectra of Mg-Li alloy specimens immersed in 3.5% NaCl solution for varying durations of zinc immersion. the capacitive reactance arc consistently shifts upward and to the right from 1 to 5 min, but at the 10-min mark, it reverses its shift and moves downward and to the left. This implies that the capacitive reactance arc initially increases and then decreases within the 1- to 10-min timeframe, reaching its minimum at 1 min. At this point, the zinc immersion time is very brief, resulting in incomplete deposition of Zn^2+^ ions and local areas without zinc coverage. When the maximum capacitive arc is reached at 5 min, it indicates that the solution resistance and charge transfer resistance are at their greatest levels. This suggests the formation of a dense zinc dip layer that effectively covers the Mg-Li alloy substrate. In comparison to a previous experimental study [25], the capacitive arc has increased, and the dissolution resistance and charge transfer resistance at 5 min have improved. This indicates an enhanced effect of zinc impregnation on the surface in this experiment. Figure 12c demonstrates the relationship between the radius of the capacitive arc resistance and the zinc content in a 3.5 wt.% NaCl solution. As the zinc content increases from 20 g/L to 50 g/L, the radius of capacitive arc resistance grows. However, as the content continues to increase beyond 50 g/L, the capacitive arc resistance gradually decreases. This suggests that the coverage of the Mg-Li alloy surface diminishes as the zinc content increases. At a zinc content of 50 g/L, the radius of the capacitive arc resistance reaches its maximum, indicating a greater resistance to charge transfer and better corrosion resistance at this content. Conversely, when the zinc content rises to 200 g/L, the radius of the capacitive arc resistance becomes the smallest, and the resistance to charge transfer reduces, indicating decreased protection of the substrate surface. Figure 12d explores the effect of temperature on the EIS. It reveals that the capacitive arc resistance is optimal at temperatures ranging from 20 to 30 °C. However, as the temperature increases to 50 °C, the radius of the capacitive arc resistance gradually decreases. In contrast to the variation observed with time and zinc content, the changes in radius are less significant, proposing that the effect of temperature on corrosion behavior is relatively limited.

### 3.4. Test of Binding Force

The assessment of electroless plating bond strength involves two tests: the file test and the scratch test. The scratch test utilizes a 30° acute angle hard knife to draw two parallel lines, spaced 2 mm apart or within a 1 mm square grid. This enables observation of any tilting or detachment of the coating from the metal matrix between the lines. On the other hand, the file test involves scraping the electroplating layer at a 45° angle from the substrate using a blunt knife. The scraping motion aligns with the grinding direction. A qualified result is achieved when the electroplating remains intact and does not peel or detach upon complete exposure to the substrate and the cross-section of the electroplating. SEM images are compared to further analyze the impact of different durations on the surface of the Mg-Li alloy. Hence, this study focuses on examining the influence of varying durations on the binding force of zinc coating. 

The file test and scratch test results were utilized to evaluate the bonding of the plated layers on different zinc-dipped Mg-Li alloys after undergoing the same post-treatment procedure. The findings presented in Table 5 show that the best bonding was obtained with a zinc dipping time of 5 min.

## 4. Conclusions

(1) Electroless-deposited zinc follows a process of the nucleation and growth of zinc grains. As the zinc dipping content, time, and temperature increase, the zinc grains gradually cover the surface of the substrate. However, the size of the zinc grains increases while the uniformity of the zinc dipping layer decreases.
(2) This study employed a galvanic solution containing a sodium-gluconate-based complexing agent for the immersion coating of Mg-Li alloys. The most densely packed and homogeneous zinc impregnation layer was obtained with a zinc content of 50 g/L, an immersion plating time of 5 min, and a temperature of 30 °C. This layer effectively covered the Mg-Li alloy substrate. Among the variables tested, the dipping time had the greatest influence on zinc dipping, while the dipping temperature had the least significant impact.
(3) Zinc dipping improves the corrosion resistance of the Mg-Li alloy by increasing the corrosion potential and reducing the corrosion current to some extent. The capacitive arc of the chemically plated layer obtained after zinc dipping increases with zinc content, time, and temperature until reaching a maximum value, after which it starts to decrease. Therefore, the optimal bond between the plated layer and the substrate was achieved with a zinc content of 50 g/L, a zinc dipping time of 5 min, and a temperature in the vicinity of 30 °C. This resulted in improved self-corrosion potential, current, capacitive arc, and surface coverage compared to previous experiments. These findings lay the groundwork for subsequent treatments of Mg-Li alloys, as zinc deposition on the surface of magnesium-lithium alloys exhibits a certain protective effect and facilitates further treatment of such alloys.

## Figures and Tables

**Table 1:** Chemical composition of each treatment.

Alkaline Degreasing (g/L)	NaOH (AR, 95%)	18
	Na_3_PO_4_ (AR, 98%)	25
	Na_2_SiO_3_ (SiO_2_, 44~47%)	7
Acid pickle (g/L)	CrO_3_ (AR, 99.99%)	150
	Fe(NO_3_)_3_ (AR, 99%)	35
	NaF (99.99% metals basis)	3.5
Activation	H_3_PO_4_(AR, ≥85 wt.% in H_2_O)	50 mL/L
	NH_4_HF (AR, 98.5%)	90 g/L
Zinc leaching (g/L)	ZnSO_4_·7H_2_O(AR, ≥99.5%)	50
	HOCH_2_(CHOH)_4_COONa (AR, 99%)	120
	C_4_H_4_O_6_KNa·4H_2_O (AR,99%)	50
	Na_2_CO_3_ (AR, ≥99.5%)	5
	LiF (AR, 99%)	3

**Table 2:** Fitting results of potentiodynamic curves in 3.5 wt.% NaCl solution in Figure 9.

Immersion Time Min	*E_∞rr_*, V	*I_∞rr_, ×*10^−6^ A/cm^2^	*Ba,* mV	*Bc,* mV
uncoated substrate	−1.63	18.64	149.44	256.12
1	−1.44	10.19	389.6	274.93
3	−1.44	5.53	407.04	260.73
5	−1.39	4.07	491.25	337.32
10	−1.63	5.75	229.846	259.21

**Table 3:** Fitting results of the potentiodynamic curves in 3.5 wt.% NaCl solution in Figure 10.

Zn Content, g/L	*E_∞rr_*_,_V	*I_∞rr_, ×*10^−6^ A/cm^2^	*Ba*, mV	*Bc*, mV
Uncoated substrate	−1.63	18.64	149.44	265.12
20	−1.43	3.96	200.45	348.4
50	−1.38	2.78	474.92	268.26
100	−1.45	5.03	326.85	261.6
200	−1.53	8.92	145.71	266.1

**Table 4:** Fitting results of the potentiodynamic curves in 3.5 wt.% NaCl solution in Figure 11.

Immersion Temperature, °C	*E_∞rr_*, V	*I_∞rr_, ×*10^−6^ A/cm^2^	*Ba,* mV	*Bc,* mV
Uncoated substrate	−1.63	18.64	149.44	265.12
20	−1.42	3.52	237.19	300.07
30	−1.40	2.82	360.41	430.06
50	−1.44	5.01	302.1	248.14
70	−1.48	5.32	213.42	235.01

**Table 5:** Binding force test results.

Zinc Dipping Time, min	File Test	Scratch Test
1	Completely detached	Peeling, not falling off
3	Peeling, not falling off	Neither blistering nor peeling
5	Neither blistering nor peeling	Neither blistering nor peeling
10	Peeling, not falling off	Peeling, not falling off

## Data Availability

All data that support the findings of this study are included in the article.
